# Protection of Ivabradine Combined with Trimetazidine on Myocardial Injury after Percutaneous Coronary Intervention in Patients with Coronary Artery Disease Evaluated by Magnetic Resonance Image under Convolutional Neural Network

**DOI:** 10.1155/2021/3150938

**Published:** 2021-09-18

**Authors:** Chunhua Chen

**Affiliations:** Department of Cardiovascular Medicine, The First People's Hospital of Chun'an County, Hangzhou 311700, China

## Abstract

**Objective:**

To evaluate the myocardial protection of Ivabradine (IBD) combined with Trimetazidine (TMZ) in patients with coronary artery disease (CAD) after percutaneous coronary intervention (PCI), magnetic resonance imaging (MRI) images under convolutional neural network (CNN) algorithm were used.

**Methods:**

A CNN artificial intelligence algorithm was proposed to process the image artifacts caused by undersampling magnetic resonance, so as to be used in the diagnosis and efficacy evaluation of myocardial injury. 120 patients with CAD after PCI were rolled into group *A* (TMZ treatment), group *B* (IBD treatment), and group *C* (IBD + TMZ combined treatment) randomly, with 40 patients in each group. All the patients were treated for two consecutive weeks and followed up for six months. Clinical indicators of patients in the two groups were observed, detected, and statistically analyzed.

**Results:**

The accuracy, sensitivity, specificity, and area under the curve (AUC) of MRI images based on CNN algorithm for the diagnosis of myocardial injury were 91.04%, 97.60%, 87.04%, and 96.43%, respectively. After treatment, the left ventricular end diastolic diameter (LVEDD), LVE diastolic volume (LVEDV), LVE systolic diameter (LVESD), and LVE systolic volume (LVESV) were greatly reduced in all groups after treatment, whereas the left ventricular ejection fraction (LVEF) increased considerably (*P* < 0.05). LVEDD, LVEDV, LVESD, and LVESV in group *C* were substantially inferior to those in groups *A* and *B*, and LVEF was remarkably superior to that in groups *A* and *B* (*P* < 0.05). After treatment, cTnI, hs-CRP, sICAM-1, ET-1, and MDA in three groups were greatly decreased (*P* < 0.05), while SOD was substantially increased (*P* < 0.05). After treatment, cTnI, hs-CRP, SICAM-1, ET-1, and MDA in group *C* were notably inferior to groups *A* and *B* (*P* < 0.05), while SOD was greatly higher (*P* < 0.05).

**Conclusion:**

MRI based on CNN had high application value in the diagnosis and efficacy evaluation of myocardial injury after PCI. For patients with CAD, IBD combined with TMZ after PCI can effectively play the role of anti-inflammatory and antioxidative damage and improve intradermal function.

## 1. Introduction 

The incidence of coronary artery disease (CAD) is usually related to abnormal lipid metabolism in the body [1]. Lipid substances in the blood are adsorbed on the intima of the coronary arteries, leading to hardening of coronary arteries, obstructing normal blood flow, and then causing myocardial ischemia and hypoxia [2, 3], which is a serious threat to the life of the patient. The current main method of treating the disease is percutaneous coronary intervention (PCI), which has the advantages of small trauma and quick recovery [4, 5]. PCI mainly uses catheters to dilate the stenosis of the coronary arteries, which helps to improve the symptoms of myocardial ischemia in CAD patients and reduces the mortality rate [6, 7]. Many reports pointed out that during PCI, coronary artery endothelial cells were damaged, inflammatory cells were activated, and the body released a large number of inflammatory factors, breaking the balance of oxygen-free radical production and elimination and increasing oxygen-free radical production, which causes damage to the heart muscle [8, 9]. Therefore, it is necessary to give patients effective drugs to correct their oxidative stress and other indexes after PCI [10]. Cardiac magnetic resonance can simultaneously evaluate the cardiac anatomy, blood perfusion, and tissue characteristics through imaging. It is the most ideal noninvasive examination method for myocardial diseases in etiological diagnosis and prognosis judgment of myocardial injury [11, 12]. Studies suggested that cardiac MRI is substantially more sensitive than conventional cardiac ultrasound in the diagnosis of diseases, such as myocardial injury. In addition, it is not only suitable for the diagnosis of suspected myocarditis but also for the etiological diagnosis of patients with similar acute coronary syndrome but normal coronary angiography [13, 14]. Convolutional neural network (CNN) is feedforward, which effectively reduces the complexity of the feedback network. It is also used to identify some two-dimensional graphs that are distorted without deformation, such as displacement and scaling [15, 16]. Neural network deals with nonuniformity and noise in image recognition. The representational learning ability of neural network is applied in many fields with the update of deep learning and numerical computing equipment [17, 18]. The parallelism and adaptability of the neural network make it suitable to deal with the regularization problem in the reconstruction of incomplete sampled data in MRI. Using appropriate prior constraints and regularization terms, the neural network algorithm can transform the reconstruction problem of magnetic resonance images (MRI) from small data sets into an optimization problem [19]. The nonlinear characteristics and noise insensitivity of neural network can effectively improve the resolution and get ideal reconstruction results.

Trimetazidine (TMZ) is an antimyocardial ischemia drug, which acts as a metabolic cytoprotective agent [8]. It has positive significance in improving the heart function after PCI. Ivabradine (IBD) is a kind of heart rate control drug [9], which has a certain promoting effect on the recovery of heart function in patients after PCI. However, there are few studies on the application of IBD combined with TMZ in CAD patients after PCI. Therefore, this work explored the treatment of CAD with IBD combined with TMZ after PCI and analyzed its protective effect on patients with cardiac function damage.

## 2. Research Methods

### 2.1. General Information

A total of 120 patients who received PCI in our hospital from February 2019 to September 2020 were randomly divided into group *A* (TMZ), group *B* (IBD), and group *C* (IBD + TMZ combined treatment), with 40 cases in each group. Group *C* included 21 men and 19 women, their ages ranged from 48 to 81 years (62.26 ± 9.42), and the course of disease ranged from 2 to 13 years (7.35 ± 2.08). Group *A* included 22 men and 18 women, their ages ranged from 46 to 83 years (64.61 ± 9.48), and the course of disease ranged from 2 to 14 years (7.97 ± 1.95 years). Group *B* included 20 men and 20 women, and their age ranged from 47 to 80 years (62.61 ± 8.35 years). There was no significant difference in general information between the three groups with a course of disease of 2 years to 14 years (7.97 ± 1.95) (*P* < 0.05), so there was some comparability. This study was approved by the Medical Ethics Committee, and the patient and their families understood the content and method of the study and agreed to sign the appropriate informed consent form.

- Inclusion criteria: patients meeting CAD diagnostic criteria and PCI indications; patients did not use TMZ two weeks before surgery; patients signed the informed consent.
- Exclusion criteria: patients with recent history of major surgery and trauma; patients with severe dysfunction of liver and kidney function; patients who were allergy to the study drug; patients with acute myocardial infarction; patients with IV level cardiac function; patients who were unable to tolerate the 6 min walk test (6MWT).

### 2.2. MRI Scan

The subject lied on the test table with head and feet behind and entered the center of the magnetic field. Both hands were placed naturally at the sides of the body. Then, the center of the electromagnetic coil was placed on the transverse axis facing the midpoint of the chest. When the tester was calm in breathing and thinking, the scan was carried out. The center of the coil was locked as the collection center and sent to the magnetic field center. Transverse, sagittal, and coronal images were completed by scanning. The scan was performed using a GE Signa (USA) 1.5 T MRI system. MRI sequence: Sag FSE T1W1 : TR/TE: 480/17 ms; FOV: 24 × 24 cm; layer thickness/spacing: 6/2 mm; matrix: 256 × 224; 2 NEX; and Sat : SI; V : S/I.

### 2.3. MRI Image Reconstruction Based on Neural Network

When the network processes MRI image data, the processed image is regarded as a two-dimensional matrix *x*, and the image reconstruction convolution process is as follows:

(1) $$\begin{array}{c} T_{\left(x\right)}=d\left(\sum_{n=1}^{y}H_{x}+p\right). \end{array}$$

Among them, *T*(*x*) is the feature map obtained by weighting the image matrix by the convolution kernel, *H* is the convolution kernel, *p* is the bias, *n* is the square of size of convolution kernel, and *d* is the activation function. In the process of training the neural network, the backpropagation algorithm can accurately identify the optimal parameters in the network. It is currently commonly used and effective. The update rule of each layer of convolution kernel *H* is

(2) $$\begin{array}{c} H_{\alpha +1}=H_{\alpha }+\Delta H_{\alpha +1}, \end{array}$$

(3) $$\begin{array}{c} \Delta H_{\alpha +1}=\beta H_{\alpha }-\theta \sigma \nabla aH_{\alpha }. \end{array}$$

**Figure 1:**
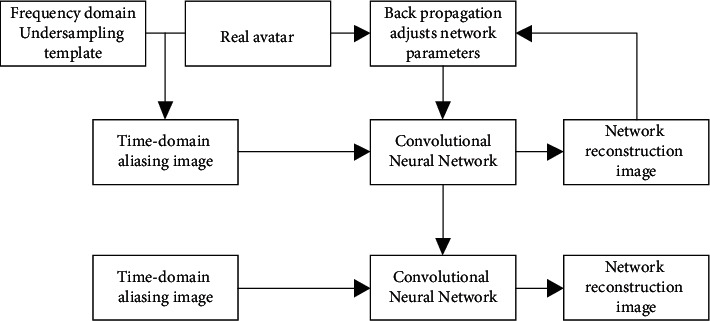
MRI image reconstruction based on neural network.

In equations (2) and (3), *H* is the convolution kernel, *α* is the number of layers, *β* is the weight of the gradient value of the previous layer, *δ* is the learning factor, *θ* is the momentum, and *a* is the loss function. The MRI image reconstruction under neural network reconstruction algorithm is presented in Figure 1.

The network parameters are adjusted through back propagation by minimizing the residual between the reconstructed image and the corresponding real image, as shown in Figure 1, which is the network training process. The optimal network is obtained by training, into which the aliasing image is input, the image reconstruction is achieved, so that the obtained high-quality image can be applied to clinical diagnosis.

Neural network has independent connection and calculation methods, and parameters are adjusted and optimized based on back propagation algorithm.

(4) $$\begin{array}{c} T\left(n\right)=\int_{-\infty }^{+\infty }I\left(x\right)O\left(n-x\right)Y_{x}. \end{array}$$

In CNN, *I*(*x*) and *O*(*x*) indicate integrable functions on *R*. Convolution of *I*(*x*) and *O*(*x*) is expressed in equation (4). *I*(*x*) is input, *O*(*x*) is convolutional kernel, and then *T*(*n*) is feature mapping.

(5) $$\begin{array}{c} T\left(n\right)=\sum_{x=-\infty }^{+\infty }I\left(x\right)O\left(n-x\right). \end{array}$$

Discrete data in images are defined as *I*(*x*) and *O*(*x*) on discrete variables *i* computer processing in practical application. Convolution of *I*(*x*) and *O*(*x*) is expressed as equation (5). Through sliding window or selective search, CNN recognizes images. It can identify and judge whether the window is the target object. The recognition is deemed as the regression problem of occurrence probability of each target in image segmentation. Convolutional layer, pooling layer, and full connection layer are how CNN is composed. The training is optimized, and parameters are updated via loss function. Convolutional layer mainly realizes the network to data convolution operation, which is the most critical infrastructure. Output signal *C* is as follows if input single sample data vector is *x*_*i*_^*a*^=[*x*_1_, *x*_2_,…, *x*_*m*_].

(6) $$\begin{array}{c} C_{i}^{t,n}=f\left(d_{n}^{t}+\sum_{s=1}^{S}h_{s}^{n}•x_{i+s-1}^{an}\right). \end{array}$$

In equation (6), *t* is the index of the layer, *f* is the activation function, which introduces nonlinear processing to the layer, *d* is deviation term of *n*th feature map, *S* is size of the convolution kernel, and *h*_*s*_ is the weight value of *n*th feature map and *s*-th filter.

Pooling includes maximum pooling and average pooling. The maximum pooling is presented in equation (7), and average pooling is illustrated in equation (8):

(7) $$\begin{array}{c} P_{i}^{t,n}=\underset{k\in K}{\max}C_{i\times L+k}^{t,n}, \end{array}$$

(8) $$\begin{array}{c} P_{i}^{t,n}=\frac{1}{\left|K\right|}\sum_{k\in K}C_{i\times L+k}^{t,n}. \end{array}$$

Equations (7) and (8) are maximum pooling and average pooling, respectively. *K* is size of pooling window, *L* is pooling step size, *C* is output signal, and *t* is the index of the layer.

The fully connected layer connects each neuron in output and upper input layers to perform feature integration, which is as follows:

(9) $$\begin{array}{c} Q_{i}^{t,n}=f\left(d_{n}^{t}+g_{i}^{t,n}•P_{i}^{t,n}\right). \end{array}$$

In equation (9), *g* is weight of fully connected layer.

To solve the problem of unclear edges and insufficient local contrast in images, adaptive histogram equalization (HE) method was used to enhance contrast of image:

(10) $$\begin{array}{c} f\left(n\right)=\frac{255\times C\left(n\right)}{X*X}. \end{array}$$

Sliding window size is *X∗X*, *C*(*n*) is cumulative distribution function, and *f*(*n*) is local mapping function. Then, slope of the local mapping function *f*(*n*) is as follows:

(11) $$\begin{array}{c} K=\frac{\text{d}\left(f\left(n\right)\right)}{\text{d}i}=\text{HE}\left(n\right)\times \frac{255}{X*X}. \end{array}$$

The maximum histogram height is *L*_max_ if the maximum local mapping function slope is *K*_max_.

(12) $$\begin{array}{c} L_{\max}=K_{\max}\times \frac{X*X}{255}, \end{array}$$

(13) $$\begin{array}{c} O_{n}=T\left(\sum_{m=1}^{t}Q_{nm}I_{n}-\gamma_{n}\right). \end{array}$$

The neural network-MP mode is presented in equation (10), where *I*_*n*_ (*n* is 1, 2, 3,…, *n*) is the input signal, and *O*_*n*_ is output of the neuron. *Q*_*nm*_ is connection strength between two neurons, *γ*_*n*_ is threshold for activating the neuron, and *T* is neuron activation function.

(14) $$\begin{array}{c} Wei\left(T,F\right)=\sum_{n=1}^{m}W_{n}f\left(T_{n},F_{n}\right). \end{array}$$

**Figure 2:**
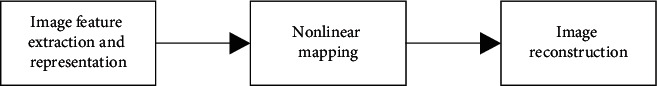
Image reconstruction process of superresolution reconstruction algorithm.

All levels of weight values are assigned by weighted loss function, which is calculated as equation (14), where *F* is predicted value input, *n* is selected sample size, *f* is loss function, and *T* is real value input. The superresolution reconstruction algorithm can realize the superresolution reconstruction of MRI images. The whole reconstruction process has three steps, and the process is shown in Figure 2.

### 2.4. Therapeutic Regimen

Both groups were treated with CAD PCI surgery, and routine secondary prevention treatments were given after PCI, including clopidogrel, aspirin, statins, angiotensin-converting enzyme inhibitors, *β*-receptor blockers, and health education. Based on the above treatments, the control group was treated with TMZ (China Ruiyang Pharmaceutical Co., Ltd.), 20 mg/time, three times a day. The observation group was treated with TMZ and IBD (Les Laboratoires Servier Industrie), 5 mg/time, 2 times a day. Treatments lasted for two weeks and followed up for six months.

### 2.5. Observation Indexes

- The cardiac ultrasound was employed to detect the heart function indexes of the two groups before and after treatment: left ventricular end diastolic diameter (LVEDD), LVE diastolic volume (LVEDV), LVE systolic diameter (LVESD), LVE systolic volume (LVESV), and LV ejection fraction (LVEF).
- Serum factors: before/after treatment, 3 mL fasting venous blood was drawn from the patient. Cardiac troponin I (cTnI) and hypersensitive C-reactive protein (hs-CRP) were detected by chemiluminescence assay. Plasma soluble intercellular adhesion molecule-1 (sICAM-1) and endothelin-1 (ET-1) were detected via enzyme-linked immunosorbent assay. The kit was from Shanghai Xinyu Biological Engineering Company, China. The content of malondialdehyde (MDA) was detected by chemical colorimetry, the superoxide dismutase (SOD) activity was detected by the xanthine oxidase method, and the kits were purchased from Beckman Coulter, USA.
- Major adverse cardiac events (MACE): the occurrence of angina pectoris recurrence, severe arrhythmia, heart failure, nonfatal myocardial infarction, and in-stent restenosis (ISR) were recorded within 6 months after surgery.
- 6 min walking test (6MWT) and Seattle Angina Questionnaire (SAQ): before and after treatment, patients were instructed to walk back and forth in a 50 m straight corridor, and the walking distance within 6 minutes was recorded. The SAQ score was measured and recorded before/after treatment, and each evaluation was made once.

### 2.6. Efficacy Evaluation Criteria

Referring to “China Common Cardiovascular and Cerebrovascular Diagnosis and Treatment Guide,” significant effect indicated that clinical symptoms and signs basically disappeared, ECG returned to normal, and cardiac function improvement level was ≥II or cardiac function grade I; effective indicated that the clinical symptoms and signs were relieved, the electrocardiogram results basically returned to normal, and the improvement of cardiac function was ≥I, but not up to I; ineffective indicated that clinical symptoms and signs, ECG results, and cardiac function improvement were not obvious, even aggravation.

### 2.7. Statistical Methods

SPSS 20.0 was employed for processing. Mean ± standard deviation was the form that measurement data were illustrated, and *t*-test was adopted. Counting data were tested by *χ*^2^ test. Rank sum test was adopted for grade data. *P* < 0.05 was considered statistically significant differences.

## 3. Results

### 3.1. MRI Image of Myocardial Injury and Denoising

**Figure 3:**
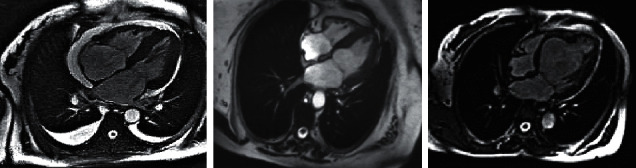
MRI images of patients with myocardial injury.

**Figure 4:**
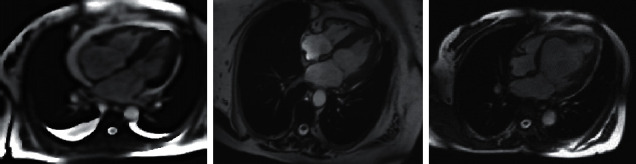
CNN denoised MRI images.

Figure 3 shows the MRI images of patients with myocardial injury. The ventricular wall thickness of patients with myocardial injury was uniform. The left ventricular dilation showed spherical change, and the motor function of bilateral ventricular wall decreased diffusely.  Figure 4 shows the image after the image artifacts were processed and denoised by undersampling MRI of patients with myocardial injury by CNN algorithm. Through the comparison of the MRI influence before and after denoising, the CNN algorithm can preserve the image details well, and the denoising effect was obvious.

### 3.2. Analysis of the Diagnosis Effect of Myocardial Injury

**Figure 5:**
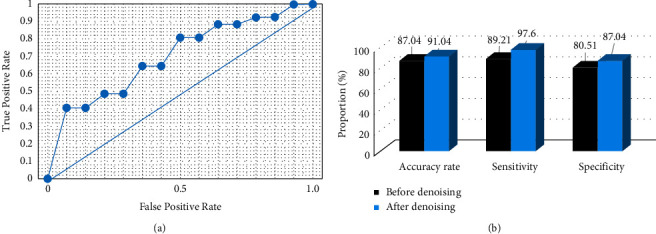
Analysis of diagnosis effect. Note. (a): the ROC curve; (b): the accuracy analysis result.

The CNN algorithm was used for the artifact processing and denoising of MRI images and then applied to the diagnosis of myocardial injury.  Figure 5 shows the results of the diagnosis effect analysis. The diagnostic accuracy, sensitivity, specificity, and area under the curve (AUC) were 91.04%, 97.60%, 87.04%, and 96.43%, respectively.

### 3.3. Clinical Efficacy

Effective rate of treatment in the IBD + TMZ group was substantially superior to TMZ group (*P* < 0.05).  Table 1 shows the details.

### 3.4. Comparison of Left Heart Function before and after Treatment

Before treatment, the LVEDD, LVEDV, LVESD, LVESV, and LVEF were compared between the groups, and differences were proved to be considerable (*P* < 0.05). After treatment, the LVEDD, LVEDV, LVESD, and LVESV of the two groups were substantially lower in contrast to that before treatment, whereas LVEF was notably increased (*P* < 0.05). The LVEDD, LVEDV, LVESD, and LVESV of IBD + TMZ group was greatly lower in contrast to that of the TMZ group, whereas the LVEF was greatly higher in contrast to that of the TMZ group (*P* < 0.05).  Table 2 shows the details.

### 3.5. Serological Index Comparison before and after Treatment

There were no considerable differences in the serum indexes before treatment between the groups (*P* < 0.05). cTnI, hs-CRP, sICAM-1, ET-1, and MDA of the two groups were substantially lower in contrast to before treatment (*P* < 0.05), but SOD was remarkably increased (*P* < 0.05). After treatment, cTnI, hs-CRP, sICAM-1, ET-1, and MDA in the IBD + TMZ group were obviously inferior to TMZ group (*P* < 0.05), whereas SOD was notably higher in contrast to that in the TMZ group (*P* < 0.05).  Table 3 shows the details.

### 3.6. MACE Occurrence

After treatment and during follow-up, the cumulative incidence of MACE in the IBD + TMZ group was remarkably inferior to that in the TMZ group (*P* < 0.05).  Table 4 shows the details.

### 3.7. Comparison of 6MWT and SAQ Scores before and after Treatment

Before treatment, there was no considerable difference in the 6MWT walking distance and SAQ score between the two groups of patients. In contrast to that before treatment, the 6MWT walking distance of the two groups of patients after treatment increased remarkably (*P* < 0.05), and the SAQ score also increased (*P* < 0.05).  Table 5 shows the details.

## 4. Discussion

CAD mostly occurs in the elderly, and PCI is often used for treatment to improve the survival rate. Moreover, the occurrence of myocardial injury after PCI seriously affects the benefits of patients, including NR, slow blood flow, and IRI [20]. PCI can induce and aggravate the local inflammatory response of coronary arteries and can activate the production of oxidative stress factors, which is not conducive to the prognosis [21]. PCI mainly uses catheters to dilate the stenosis of the coronary arteries, which improves the symptoms of myocardial ischemia in CAD patients and reduces the mortality rate. During PCI, coronary artery endothelial cells were damaged, inflammatory cells were activated, and the body released massive inflammatory factors, breaking the balance of oxygen-free radical production and elimination, and increasing oxygen-free radical production, which causes damage to the heart muscle. Therefore, it is imperative to give patients effective drugs to correct their oxidative stress and other indexes after PCI. Cardiac magnetic resonance, as a noninvasive and nonradiation efficient examination method, has high resolution and good contrast, and it is widely used in clinical practice. Delayed enhancement technique is used to detect small myocardial injury, which is crucial for the diagnosis of myocardial diseases [22]. CNN artificial intelligence algorithm was proposed to process the image artifacts caused by undersampling magnetic resonance, which was used in the diagnosis and efficacy evaluation of myocardial injury. The results showed that the CNN algorithm can preserve the image details well, and the denoising effect was obvious. The accuracy, sensitivity, specificity, and AUC of MRI were 91.04%, 97.60%, 87.04%, and 96.43%, respectively. It may be that the CNN improved the recognition efficiency and accuracy of MRI images of myocardial injury after reconstruction and artifact removal, thus improving the diagnostic effect. TMZ protects cells by directly inhibiting enzymes in long mitochondrial chains, which can ensure the normal function of ion pumps and permeable membrane sodium-potassium flow. Moreover, it can play the role of antioxidation, scavenging oxygen-free radicals and inhibiting neutrophils aggregation. IBD mainly inhibits If current and slows heart rate by selectively blocking If channels of P cells in the sinoatrial node. However, there are few studies on the application of IBD combined with TMZ in CAD patients after PCI. Therefore, this work explored the treatment of CAD with IBD combined with TMZ after PCI and analyzed its protective effect on patients with cardiac function damage.

The results suggested that the serum cTnI, hs-CRP, sICAM-1, ET-1, and MDA in the IBD + TMZ group were greatly reduced, and the serum SOD content was greatly increased. The efficacy was better in contrast to that of patients in the TMZ group, showing that it helped maintain the balance of oxygen-free radical production and clearance in the body, relieved myocardial damage caused by inflammation and lipid peroxidation, and had a protective effect on heart function. The adoption of IBD after PCI helped to reduce the expression of myocardial injury markers. The improvement of LVEDD, LVEDV, LVESD, LVESV, LVEF, and 6 min walking distance in the IBD + TMZ group was also superior to TMZ group. In addition, the total effective rate of clinical curative effect in the IBD + TMZ group was remarkably superior to that of the TMZ group, which further showed that the combination of drugs can reduce the inflammatory response after PCI, reduce oxidative damage and vascular endothelial damage, help promote the recovery of left heart function, and improve clinical efficacy.

Further studies showed that patients in the IBD + TMZ group walked more in contrast to the TMZ group at 6 minutes after treatment, and the SAQ score was higher in contrast to that of the TMZ group. It was suggested that the combination of IBD and TMZ can alleviate symptoms and improve patients' mobility and quality of life. During the follow-up, the cumulative incidence of MACE in the IBD + TMZ group was inferior to that in the TMZ group, indicating that the combination of IBD and TMZ had continuous anti-inflammatory and antioxidant damage, and improved intradermal function, thereby reducing the occurrence of MACE after PCI and improving the prognosis of patients.

In conclusion, MRI image based on CNN algorithm have high application value in the diagnosis and efficacy evaluation of myocardial injury after PCI. For patients with CAD, IBD combined with TMZ after PCI can effectively play the role of anti-inflammatory and antioxidative damage and improve intradermal function.

## Figures and Tables

**Table 1:** Contrast of clinical efficacy.

Group	Significant effect (*n*)	Effective (*n*)	Ineffective (*n*)	Effective rate (%)
IBD + TMZ	20	15	5	87.50
TMZ	13	14	13	67.50
*P*		-		0.012

**Table 2:** Contrast of left heart function before and after treatment between the two groups.

Index		IBD + TMZ group	TMZ group
LVEDD (mm)	Before	61.97 ± 4.07	62.06 ± 3.95
	After	40.88 ± 3.17^*∗*^#	49.16 ± 3.42^*∗*^

LVEDV (mL)	Before	112.97 ± 8.96	113.07 ± 9.42
	After	91.14 ± 6.21^*∗*^#	101.20 ± 7.04^*∗*^

LVESD (mm)	Before	43.14 ± 3.86	42.88 ± 4.16
	After	32.18 ± 3.51^*∗*^#	37.62 ± 3.66^*∗*^

LVESV (mL)	Before	65.21 ± 5.38	65.42 ± 5.69
	After	40.91 ± 3.79^*∗*^#	50.28 ± 4.42^*∗*^

LVEF (%)	Before	41.08 ± 4.15	41.15 ± 4.50
	After	54.83 ± 5.02^*∗*^#	49.14 ± 4.93^*∗*^

*Note.* In contrast to before treatment within group, ^*∗*^*P* < 0.05; in contrast to TMZ group in the same period, #*P* < 0.05.

**Table 3:** Contrast of cTnI, hs-CRP, MDA, and SOD before and after treatment.

Index		IBD + TMZ group	TMZ group
cTnI (ng/mL)	Before	1.71 ± 0.26	1.70 ± 0.27
	After	0.42 ± 0.05^*∗*^#	0.97 ± 0.08^*∗*^

Hs-CRP (mg/L)	Before	16.27 ± 1.73	16.31 ± 1.82
	After	7.17 ± 0.42^*∗*^#	11.50 ± 1.01^*∗*^

sICAM-1 (mg/L)	Before	263.14 ± 36.63	259.37 ± 31.18
	After	158.81 ± 21.55^*∗*^#	216.59 ± 25.65^*∗*^

MDA (*µ*moL/L)	Before	8.56 ± 1.16	8.53 ± 1.21
	After	4.37 ± 0.38^*∗*^#	6.83 ± 0.68^*∗*^

SOD (U/mL)	Before	62.27 ± 5.97	63.17 ± 6.08
	After	88.94 ± 9.17^*∗*^#	71.26 ± 8.85^*∗*^

ET-1 (ng/L)	Before	46.65 ± 6.22	47.06 ± 6.15
	After	35.73 ± 5.82^*∗*^#	43.08 ± 5.96^*∗*^

*Note.* In contrast to before treatment within group, ^*∗*^*P* < 0.05; in contrast to TMZ group in the same period, #*P* < 0.05.

**Table 4:** Comparison of MACE occurrence.

Group	Angina recurrence (*n*)	ISR (*n*)	Severe arrhythmia (*n*)	Heart failure (*n*)	Nonfatal myocardial infarction (*n*)	Cumulative incidence (%)
IBD + TMZ	5	3	1	0	1	25.00
TMZ	12	6	3	1	3	62.50
*P*	—					0.009

**Table 5:** 6MWT and SAQ scores before and after treatment.

Group	Period	6MWT (m)	SAQ score
IBD + TMZ	Before	195.78 ± 30.13	64.17 ± 8.06
	After	389.81 ± 40.34^*∗*^#	83.20 ± 8.79^*∗*^#

TMZ	Before	197.37 ± 31.94	64.08 ± 8.13
	After	295.33 ± 32.17^*∗*^	93.05 ± 9.64^*∗*^

*Note.* In contrast to before treatment within group, ^*∗*^*P* < 0.05; in contrast to TMZ group in the same period, #*P* < 0.05.

## Data Availability

The data used to support the findings of this study are available from the corresponding author upon request.
